# A particle swarm optimization approach for predicting the number of COVID-19 deaths

**DOI:** 10.1038/s41598-021-96057-5

**Published:** 2021-08-16

**Authors:** Mohamed Haouari, Mariem Mhiri

**Affiliations:** Department of Mechanical and Industrial Engineering, College of Engineering, Qatar University, Doha, Qatar

**Keywords:** Applied mathematics, Epidemiology

## Abstract

The rapid spread of the COVID-19 pandemic has raised huge concerns about the prospect of a major health disaster that would result in a huge number of deaths. This anxiety was largely fueled by the fact that the severe acute respiratory syndrome coronavirus 2 (SARS-CoV-2), responsible for the disease, was so far unknown, and therefore an accurate prediction of the number of deaths was particularly difficult. However, this prediction is of the utmost importance for public health authorities to make the most reliable decisions and establish the necessary precautions to protect people’s lives. In this paper, we present an approach for predicting the number of deaths from COVID-19. This approach requires modeling the number of infected cases using a generalized logistic function and using this function for inferring the number of deaths. An estimate of the parameters of the proposed model is obtained using a Particle Swarm Optimization algorithm (PSO) that requires iteratively solving a quadratic programming problem. In addition to the total number of deaths and number of infected cases, the model enables the estimation of the infection fatality rate (IFR). Furthermore, using some mild assumptions, we derive estimates of the number of active cases. The proposed approach was empirically assessed on official data provided by the State of Qatar. The results of our computational study show a good accuracy of the predicted number of deaths.

## Introduction

In the last days of December 2019, a mysterious disease was first diagnosed in Wuhan City, Hubei Province, China. Within days, a genomic analysis revealed the disease was caused by a new coronavirus called severe acute respiratory syndrome coronavirus 2 (SARS-CoV-2)^1,2^. Shortly after, the World Health Organization (WHO) named the new disease COVID-19, and assessed the on-going outbreak as a pandemic. Since January 2020, and like a tsunami, a SARS-CoV-2 wave has rapidly spread across the world and has silently claimed in its wake the lives of millions of people. Public health authorities around the world had to face a very serious situation that was extremely complicated by the lack of important key facts related to the characteristics of SARS-CoV-2. In this article, we address three critical (related) questions that policymakers around the world have faced since the start of the pandemic. These questions are the following:

- Q1: What is the extent of the pandemic in the population?
- Q2: What is the infection fatality rate (IFR)?, which is defined as the ratio between the number of deaths due to the disease and the number of infected individuals (this rate should not be confused with the so-called case fatality rate (CFR), which is equal to the death rate of individuals who test positive).
- Q3: What is the expected number of deaths?

Clearly, answering to Q1 will provide a key to Q2 and thereby will make Q3 pretty easy. However, the former question is extremely challenging because a significant portion of positive cases are asymptomatic and therefore remain largely undetected. For instance, a recent study by Anand et al.^3^ found that in the US, the number of individuals who formed antibodies against SARS-CoV-2 (which is an evidence that they contracted the disease) represents about ten times the number of those that were tested positive. However, answering these questions is of utmost importance for public health authorities in order to design the most effective response policies to contain the epidemic, mitigate its devastating impact, and save lives. Obviously, testing an entire population for infected cases is, in most cases, out of the question. Therefore, sampling is widely used to obtain estimates of the extent of the epidemic. In particular, measures of the prevalence of SARS-CoV-2 antibodies in large population samples has been used by many authors for deriving estimates of the rate of infection^3^. In this article, we take a purely mathematical/algorithmic approach to answer the aforementioned questions. Our main goal is to derive a mathematical model to predict the number of deaths from COVID-19. Moreover, the model can be used to obtain estimates of IFR, number of infected cases and number of active cases.

In addition to its simplicity, a special feature of the proposed approach is that it relies on *parsimonious* input data. More specifically, it requires the following inputs: (1) Daily number of deaths, and (2) Minimum and maximum time elapsed from infection to death (no distribution is required). Furthermore, to obtain estimates of the number of active cases, minimum and maximum time between infection and recovery are needed as well. The basic idea is to model the total number of infected cases using a generalized logistic function (also called Richards’ curve) having four parameters. These parameters are estimated by minimizing the sum of the quadratic errors between the actual cumulative number of deaths and the predicted ones. An approximate solution to the optimization problem is obtained using a Particle Swarm Optimization algorithm (PSO). The effectiveness of the approach is assessed through experiments that were carried out on official data provided by the State of Qatar. The choice of this country is motivated in particular by the fact that since the detection of the first local COVID-19 positive case (on February 29, 2020), the epidemic has spread on such a large scale that Qatar has topped the world ranking for the rate of positivity in COVID-19 (which is measured as the ratio of positive cases to the total population).

## Literature review

As per the study carried out in^4^, mathematical predictive models dealing with COVID-19 can be classified into two main categories: transmission dynamics models and phenomenological models. The main purpose of the first models category is to describe the mechanisms underlying an epidemic and understand its behaviour as well as the effects of human interventions. These models use a large number of parameters unlike the second models category. Many research studies were conducted to provide a transmission dynamic model for COVID-19. In^5^, an SIR (susceptible-infected-recovered) epidemic model for COVID-19 spread in Indonesia with fuzzy parameter was constructed by taking into account the factors of vaccination, treatment, health protocols, and the virus-load. In^6^, a conceptual mathematical model on the transmission dynamics of COVID-19 between the frontliners and the general public was formulated by considering an SEIR (susceptible–exposed–infected–recovered) compartment model where a huge number of parameters is required (up to 23 parameters). According to the available infection data, authors in^7^ elaborate a dynamical model based on the fractional derivatives of nonlinear equations that describe the outbreak of COVID-19. Such a model necessitates a very large number of parameters. An epidemiological model whose implementation is made in Excel spreadsheets is developed in^8^. When adjusting many parameters, it was shown that the model follows the evolution of COVID-19. A new SIR model was developed in^9^ including direct and fomite transmission as well as household structure applied to COVID-19. The model involves many parameters such as: the infection rate related to direct transmission, the infection rate related to fomite transmission, the minimum contact radius and the average number of daily cross-household encounters between a susceptible and an infected person. In^10^, a fractional epidemic model in the Caputo sense taking into account quarantine, isolation, and environmental impacts was developed in order to track the dynamics of the COVID-19 pandemic. A Gaussian Process Regression model was proposed in^11^ to predict the number of COVID-19 deaths. It was compared to Artificial Neural Network model. The study examines the impact of age, number of smokers and number of diabetic patients on increasing the number of deaths.

Phenomenological models which rely on a limited number of parameters, can afford a quick epidemic forecast efficiently^4^. In this context, a bimodal lognormal distribution function is developed in^4^ for the prediction of the time distribution of COVID-19 deaths. Nevertheless, this model cannot be used for estimating the IFR nor the number of active cases. In^12^, a demographic scaling model for estimating the total number of COVID-19 infections was developed. This approach requires the following input data: the number of COVID-19-related deaths for the population of interest; and the age-specific infection fatality rates from a reference population, scaled to match the target population based on life tables. The study in^13^ deals with the impact of air pollution during the COVID-19 pandemic on public health where a linear regression analysis was conducted. It was demonstrated that geo-environmental conditions can speed up the spread of COVID-19 in Italy and thus contribute to higher number of infected individuals and deaths.

Numerous research studies were conducted to investigate the influence of socioeconomic, demographic, climatological and environmental factors on the spread on COVID-19^14^. The idea in^14^ consists in proposing an index quantifying the environmental risk of exposure of cities or regions to future epidemics of the COVID-19 and similar pandemics. Such an index helps policymakers in preventing future epidemics. In^15^, the study investigates correlation of the spread and decay durations with population density, temperature, and absolute humidity in four countries. In^16^, the study shows how the duration of lockdownd due to the COVID-19 pandemic, has an impact on the rates of infected people and deaths, as well as the growth of Gross Domestic Product (GDP) of nations. A comparative analysis of the first and second wave of COVID-19 was made in^17^. This study helps in establishing adequate decisions regarding the public health versus next COVID-19 waves or other pandemics. The approach of^18^ analyzes the impact of socio-economic factors on the reduction of the COVID-19 fatality rate. It suggests that high GDP per capita, high healthcare spending and low levels of air pollution can reduce the fatality rate. An estimation study of the dispersion parameter was developed in^19^. This parameter helps in measuring the heterogeneity in COVID-19 transmission. The distribution of the number of secondary cases is considered as a negative binomial (NB) distribution with the dispersion parameter. In^20^, an estimation study of the excess of deaths in the United States due to the COVID-19 pandemic from March to May 2020 was conducted. The approach was based on data on all-cause deaths and pneumonia/influenza/COVID-19 deaths. An estimation study of age and sex-specific excess mortality risk and deaths due to COVID-19 was elaborated in^21^. The study involves the estimation of life expectancy at birth and lifespan inequality too.

Unlike the aforementioned studies, our approach describes a novel phenomenological model that enables to derive an estimate of the number of positive cases (including the undetected cases) along with the IFR. Our work is in line with many mathematical approaches developed so far to model various epidemics such as severe acute respiratory syndrome (SARS)^22–24^, Ebola^25^, Middle East respiratory syndrome (MERS)^24,26^, etc. Indeed, the authors of^22^ proposed a simple mathematical model to predict the number of cases and deaths of SARS. Unlike the algorithmic estimation methods, the developed model consisted in trying out several values of the parameters enabling the best fitting that matches the observed data. This modelization is considered simple to help the front line public health practitioners. In^25^, the study was about modeling the transmission dynamics of Ebola virus disease in Liberia and estimating the basic reproduction number when there is no control measures. It has been shown that the epidemic can be well contained if isolation and safe burial measures are taken. The provided results help in establishing guidance and allow the spread control of the virus. A compartmental model for the outbreak of MERS similar to SARS models was studied in^26^. A coupled system of non linear ordinary differential equations was considered. The study showed that the contact coefficients, the isolation rate constant and the transmission factor have a huge impact on the containment time and the outbreaks severity. In^23^, an SEIJR model (susceptible, exposed, infected, diagnosed, recovered) is considered for the study of SARS epidemic by including diffusion in the system in order to examine the spacial spread of the disease. The diffusion impact on the disease spread was investigated for different initial population distributions and interventions effect was analyzed. Authors in^27^ modeled the interaction between the epidemic spreading and information diffusion by developing a two-layer network. The study showed that the knowledge diffusion has a significant impact on controlling not only rumor but also epidemic. The purpose of^28^ consisted in developing a global threshold dynamics for several SVEIS (susceptible, vaccinated, exposed, infectious) epidemic models with temporary immunity, vaccination, latency and nonlinear incidence. The study revealed that reducing the outbreak peaks (such as: global seasonal influenza burden, measles cases surge and COVID-19 pandemic) can be efficient and ensured with timely vaccination more than other measures.

Since the first weeks of the COVID-19 epidemic, multiple research studies were elaborated aiming at predicting the new virus evolution and dynamics in order to guide public health authorities to take the most reliable decisions to protect people. A comparison study of the epidemics spread has been elaborated in^24^ between the new virus COVID-19, SARS and MERS. A propagation growth model was established for the three epidemics and a nonlinear fitting was considered for the determination of their parameters. The obtained results confirmed that the COVID-19 presents the highest growth rate and the shortest multiplication cycle versus SARS and MERS. Authors in^29^ investigated two models to predict the COVID-19 confirmed cases in the most affected countries: the autoregressive integrated moving average (ARIMA) and the least square support vector machine (LS-SVM). It has been concluded that the LS-SVM contributes to more accuracy than the ARIMA model. The provided predictions enable authorities to take the most convenient decisions and precautions. In^30^, the main idea consisted in predicting the number of positive cases of the COVID-19 pandemic in Italy with time evolution. The mathematical model is based on official data as well as on the use of a cumulative distribution function by referring to the Gauss error function with four parameters. The proposed approach provided very good fit with the China case when plotting the cumulative number of cases and the cumulative number of fatalities. The number of positive cases in Italy is then predicted by performing a number of fits of the available data. The fit flex point and the day in which a substantial attenuation of daily cases are both determined. The latter results were confirmed by performing Monte-Carlo simulations. Authors in^31^ provided estimating models to characterize the dynamics of SARS-CoV-2 transmission in France. Delays from hospitalization to death and from hospitalization to ICU (Intensive Care Unit) are estimated by age range according to collected data. The infection fatality ratio is calculated from the Diamond Princess and the age distribution of the French hospitalized population. The risk of hospitalization, ICU admission and death are modeled and the impact of the lockdown has been taken into account to compute the transmission rate. Authors in^32^ developed prediction models based on Genetic Programming to estimate confirmed cases (CC) and death cases (DC) of COVID-19 in three states of India as well as whole India. The gene expression programming model used simple linkage function and provided robust and reliable results (for CC and DC). It relied on daily situation reports of COVID-19 published by the Indian government. It was shown that proposed models are less sensitive to variables, based on experimental data and can be used to predict future cases. The expression trees simplicity introduced basic mathematical equations that can be implemented without time consuming. The main idea of^33^ consisted in estimating the COVID-19 evolution in terms of cumulative number of infected cases, daily infected cases and the Fisher–Pry. The predictive model was based on the SIR model (SIR stands for Susceptible, Infected and Recovered) reduced to the Verhulst equation. It provided a reliable and accurate prediction of the epidemic for a whole country. According to the Fisher–Pry plots for different countries, the SIR-Verhulst modelization proved its efficiency in quantifying public health measures. That’s why relying only on such a plot can be sufficient to reveal significant conclusions about the epidemic evolution in different countries.

In^34^, an estimation of the time between symptoms onset and outcome (death or recovery) is made based on individual-case data for infected cases dying due to COVID-19 in Hubei, Wuhan. This parameter was estimated as 17.8 days from onset-to-death and as 24.7 days from onset-to-recovery. Overall case fatality ratio (CFR) in China was estimated as 13.8% while the estimated overall infection fatality rate (IFR) was 0.66%. Authors provided detailed estimates of the two latter parameters for different age groups and showed higher ratios estimates for older persons (CFR: 13.4% and IFR: 7.8% for persons aged more than 80 years-old). In^35^, authors developed a modified SIR model to predict COVID-19 evolution in Italy. The idea consisted in considering the initial number of susceptible individuals as well as a proportionality between the detected number of positive cases and the actual/unknown number of infected individuals. The model parameters were determined for many Italian regions using a standard weighted least-squares optimization problem. The main idea in^36^ consisted in developing a prediction model of COVID-19 enabling a good fitting of data with a high regression coefficient and predicting the total infections and the day of the infection peak. The model provided reliable predictions for China, Italy and Spain. The impact of partial/total lockdown on the pandemic spread was studied through the curves interpretation and it was concluded that the epidemic lifespan is lockdown independent. Authors in^37^ proposed a generalized SEIR (susceptible, exposed, infectious, recovered) model including insusceptible cases, exposed cases and quarantined cases in order to predict inflection point and possible ending time of the COVID-19 in 24 provinces in Mainland of China and 16 counties in Hubei province. The considered model involved several key parameters that were estimated as well such as: latent time and quarantine time. The model provided reliable results fitting the empirical public data. Authors in^38^, developed a new mathematical SEIR-HC [SEIR (susceptible, exposed, infectious, removed), HC (Hospital, Community)] model by considering two different social circles (individuals in hospital and community). A two-step iterative optimization approach is proposed to estimate the model parameters. It has been shown that quarantine and isolation are important measures that play a considerable role in reducing the epidemic spread. A mathematical model for COVID-19 was developed in^39^ taking into consideration the impact of imported cases and different rates (isolating, diagnostic, recovery, mortality). The model is dynamic for the epidemic transmission process in terms of quantity of daily infected individuals. The results showed that the isolation of infected cases and the strict control of input cases contribute in stopping the epidemic spread. Authors in^40^ developed a SEIQR (susceptible, exposed, infected, quarantined, recovered) mathematical model to study the COVID-19 spread. The model is based on nonlinear system of coupled ordinary differential equations. A study of the model in terms of consistency, stability and convergence was carried out and showed its validity. A two-parameter SIR mathematical model is investigated in^41^ to study the SARS-Cov-2 epidemic. The elaboration showed that the SIR model is restricted as it can be applied reliably on small populations to provide consistent results.

## Optimization approach for COVID-19 deaths estimation

In the sequel, we shall use the following notation.

- *Input data*
  - [1, *T*]: Time interval during which observations on deaths were reported,
  - $\tilde{D}(t)$ : Reported number of new deaths on day *t*, $t = 1, \ldots ,T$,
  - $\tilde{C}_d(t) = \sum \limits _{k=1}^{t} \tilde{D}(k)$: Reported cumulative number of deaths on day *t*, $t = 1, \ldots ,T$,
  - $\tau _{\min }$/$\tau _{\max }$: Minimum/Maximum time from infection to death. These values can be defined as the lower and upper bounds of the $95\%$ tolerance interval of the random time that elapses from infection to death,
  - $r_{\min }$/$r_{\max }$: Minimum/Maximum time from infection to recovery. These values can be defined as the lower and upper bounds of the $95\%$ tolerance interval of the random time that elapses from infection to recovery.

- *Estimate values*
  - *I*(*t*): Estimate of total number of cases on day *t*, $t = 1, \ldots ,T$,
  - *N*(*t*): Estimate of number of new cases on day *t*, $t = 1, \ldots ,T$,
  - *R*(*t*): Estimate of number of recovered cases on day *t*, $t = 1, \ldots ,T$,
  - *A*(*t*): Estimate of number of active cases on day *t*, $t = 1, \ldots ,T$.

The total number of cases *I*(*t*) is assumed to follow a generalized logistic function. The generalized logistic function is an S-shaped function that has been originally used for modeling growth curves. In contrast to the well-known logistic function (Verhulst function), the generalized logistic function is used for modeling situations where the growth curve is asymmetrical about its inflection point. Several variants of this function have been investigated so far. In this paper, we consider the following form:

1 $$\begin{aligned}&I(t)&\quad =&\quad 0,&\quad t < 0,\end{aligned}$$

2 $$\begin{aligned}&I(t)&\quad =&\quad \frac{K}{(1+Qe^{-\lambda t})^{1/{\nu }}},&\quad t \ge 0, \end{aligned}$$

where $(K, Q, \lambda , \nu )$ are four parameters allowing a high flexibility of the sigmoid curve. During the last years, the generalized logistic model has been widely used in epidemiology for deriving predictions of the spread of epidemics including SARS^42,43^ and dengue fever^44,45^. Recently, several authors used it for deriving prediction models for the COVID-19 outbreak^46–48^. The computation of the optimal parameters of a generalized logistic function is a challenging nonlinear least squares problem that has been addressed by many authors^49^.

### Remark

To our knowledge, all of the previously published papers dealing with the use of the generalized logistic function to predict the spread of an epidemic address the problem of fitting positive case data. However, in the case of the COVID-19 outbreak, focusing on positive cases is largely questionable. Indeed, as the number of cases infected with COVID-19 is generally much higher than the number of positive cases diagnosed, the practical relevance of the approach then becomes questionable. In contrast, in this paper, we shall build a model for predicting the total number of cases (including both detected and undetected ones).

The number of new cases on day *t* is denoted by *N*(*t*) and is deduced as follows:

3 $$\begin{aligned}N(t) = 0,\quad t \le 0, \end{aligned}$$

4 $$\begin{aligned}N(t) = I(t) - I(t-1),\quad t > 0. \end{aligned}$$

Consequently, the number of new deaths on day *t* is modeled as the the sum of a linear combination of the number of new cases of days $t-\tau _{\max },t-\tau _{\max }+1, \ldots ,t-\tau _{\min }$. Hence, we set:

5 $$\begin{aligned}D(t) \,=\,& \sum \limits _{\zeta = t-\tau _{\max }}^{t-\tau _{\min }} a_{t-\zeta } N(\zeta ),&\quad t=1, \ldots ,T, \end{aligned}$$

6 $$\begin{aligned}\, =\,& \sum \limits _{j = \tau _{\min }}^{\tau _{\max }} a_j N(t-j),&\quad t=1, \ldots ,T, \end{aligned}$$

where $a_j$ is the expected rate of individuals that die *j* days after being infected ($j=\tau _{\min }, \ldots ,\tau _{\max }$).

Accordingly, the cumulative number of deaths can be expressed as follows:

7 $$\begin{aligned}C_d(t) = \sum \limits _{k=1}^{t} D(k),\quad t=1, \ldots ,T, \end{aligned}$$

8 $$\begin{aligned} \quad \quad = \sum \limits _{k=1}^{t}\sum \limits _{j = \tau _{\min }}^{\tau _{\max }} a_j N(k-j),\quad t=1, \ldots ,T, \end{aligned}$$

Similarly to the daily deaths, the number of infected people that recover on day *t* is modeled as the the sum of a linear combination of the number of new cases of days $t-r_{\max },t-r_{\max }+1, \ldots ,t-r_{\min }$. Thus, we define:

9 $$\begin{aligned}R(t)= \sum \limits _{\eta = t-r_{\max }}^{t-r_{\min }} b_{t-\eta } N(\eta ), \quad t=1, \ldots ,T, \end{aligned}$$

10 $$\begin{aligned}= \sum \limits _{j = r_{\min }}^{r_{\max }} b_{j} N(t-j), \quad t=1, \ldots ,T, \end{aligned}$$

where $b_j$ represents the rate of individuals that recover *j* days after being infected ($j=r_{\min }, \ldots ,r_{\max }$).

Clearly, $a_j$ ($b_j$) can be seen as the probability that an individual will die (recover) *j* days after being infected. Therefore, we have:

11 $$\begin{aligned} \sum \limits _{j = \tau _{\min }}^{\tau _{\max }} a_{j} + \sum \limits _{j = r_{\min }}^{r_{\max }} b_{j} = 1. \end{aligned}$$

In the sequel, we shall make the assumption that the distribution of the $b_j$ is unimodal. We denote the mode by $j^*$. This assumption is supported by the findings of^34^ who showed that the best fit of the onset-to-recovery distribution is a gamma distribution.

Therefore, the number of active cases on day *t* is denoted by *A*(*t*) and is given by:

12 $$\begin{aligned} A(0) = I(0), \end{aligned}$$

13 $$\begin{aligned} A(t) = A(t-1) + N(t) - D(t) - R(t), \quad t=1, \ldots ,T. \end{aligned}$$

To estimate the cumulative number of deaths, we use the following regression model:

14 $$\begin{aligned} \tilde{C}_d(t) = C_d(t) + \epsilon _t, \quad t=1, \ldots ,T, \end{aligned}$$

where $\epsilon _t$ is a normally distributed random error with mean zero.

Using (8), we get:

15 $$\begin{aligned} \tilde{C}_d(t) = \sum \limits _{k=1}^{t}\sum \limits _{j = \tau _{\min }}^{\tau _{\max }} a_j N(k-j) + \epsilon _t, \quad t=1, \ldots ,T. \end{aligned}$$

Given a quadruplet $(K,Q,\lambda ,\nu )$, we define $Z(K,Q,\lambda ,\nu )$ as the optimal value of the following Quadratic Programming (QP) problem:

16 $$\begin{aligned} \text {(QP):}&Z(K,Q,\lambda ,\nu ) = \text {Min} \sum \limits _{t=1}^{T}\epsilon _t^2 \end{aligned}$$

17 $$\begin{aligned}&\quad&\sum \limits _{k=1}^{t}\sum \limits _{j = \tau _{\min }}^{\tau _{\max }} a_j N(k-j) + \epsilon _t&= \tilde{C}_d(t),&\quad t=1, \ldots ,T, \end{aligned}$$

18 $$\begin{aligned}&\quad&\sum \limits _{j = \tau _{\min }}^{\tau _{\max }}a_j+\sum \limits _{j = r_{\min }}^{r_{\max }}b_j&= 1, \end{aligned}$$

19 $$\begin{aligned}&\quad&b_{j-1}&\le b_j,&\quad j=r_{\min }+1, \ldots ,j^*,\end{aligned}$$

20 $$\begin{aligned}&\quad&b_{j}&\ge b_{j+1},&\quad j=j^*, \ldots ,r_{\max }-1,\end{aligned}$$

21 $$\begin{aligned}&\quad&a_j&\ge 0,&\quad j=\tau _{\min }, \ldots ,\tau _{\max },\end{aligned}$$

22 $$\begin{aligned}&\quad&b_j&\ge 0,&\quad j=r_{\min }, \ldots ,r_{\max }. \end{aligned}$$

Clearly, (QP) aims at finding the values of the $a_j$’s, $b_j$’s and $\epsilon _t$’s that minimize the sum of quadratic errors between the reported cumulative number of deaths and the predicted ones. Constraints (17) and (18) are self-explanatory, and constraints (19)–(20) enforce the unimodularity of the infection-to-recovery time distribution.

To find the parameters of the generalized logistic function, we formulate a Global Optimization problem (GO) that requires finding the quadruplet that minimizes *Z*(.). Hence, the problem reads as follows:

23 $$\begin{aligned} \text {(GO): Min}&Z(K, Q,\lambda ,\nu )&&\end{aligned}$$

24 $$\begin{aligned}&\quad&K, Q, \lambda , \nu \ge&0. \end{aligned}$$

Finding a global minimum of Problem (GO) poses very hard challenges. Instead, we derive an approximate solution by using a Particle Swarm Optimization algorithm (PSO). Since its introduction about two decades ago by Kennedy and Eberhart^50^, this approach has been extensively used for solving hard continuous global optimization problems (See^51–53^ and the references therein). PSO algorithm was applied in several research studies to deal with COVID-19 prediction. In^54^, a susceptible-exposed-infected-quarantined-recovered-dead (SEIQRD) model was presented to predict the COVID-19 spread in Italy. The model parameters are estimated using the PSO algorithm. In^55^, a novel PSO-BLS (particle swarm optimization broad learning system) was developed to predict the dynamics of COVID-19. The proposed model shows higher accuracy and stability versus deep learning models when predicting the number of active infected cases and removed cases. Four phenomenological epidemic models as well as SIR model were investigated in^56^ in order to predict the cumulative number of confirmed cases due to COVID-19 in Saudi Arabia and the possible end-date of the pandemic. The idea was based on an optimization approach using the PSO algorithm.

Once problem (GO) is solved, an IFR estimate is computed as the sum of the infection-to-death probabilities:

25 $$\begin{aligned} IFR = \sum \limits _{j = \tau _{\min }}^{\tau _{\max }}a_j. \end{aligned}$$

## Numerical results

We consider the following settings:

1. The minimum/maximum time from onset of symptoms to death is 5 days/21 days^34^.
2. The minimum/maximum time from onset of symptoms to recovery is 4 days/34 days^34^. The mode of this distribution is 22.
3. The mean incubation time (IT) of COVID-19 is about 6 days^57^. Hence, we set the minimum/maximum time from infection to death to 11 days/27 days, the minimum/maximum time from infection to recovery to 10 days/40 days, and the mode of the infection-to-recovery distribution to 28 days.
4. The empirical data of cumulative deaths are provided in^58,59^. The date $t = 1$ corresponds to February 29, 2020, which is the date when the first positive case was detected in Qatar.

The proposed approach was implemented via MATLAB R2019b (we used the PSO subroutine embedded therein), and run on a machine with an Intel i5-2430M processor and 6 GB of RAM.

**Figure 1:**
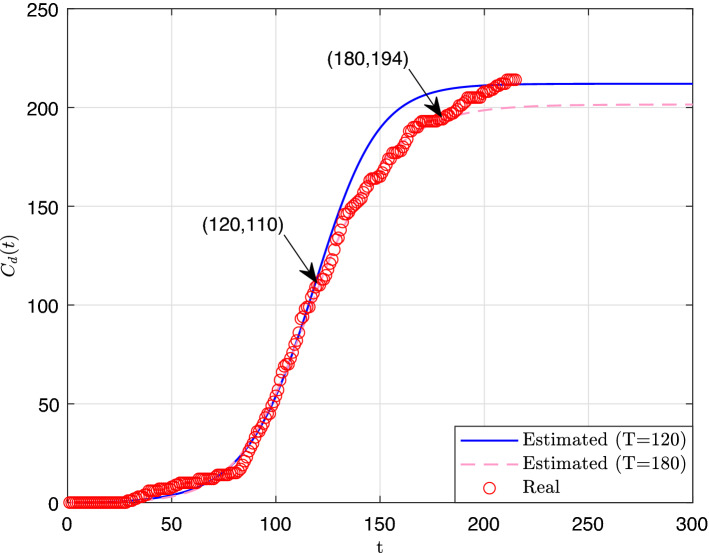
Estimate cumulative number of deaths curves.

To assess the performance of the proposed approach, we run it on two input sets that were obtained by considering the number of deaths that were reported during the first 120 and 180 days of the outbreak, respectively. The obtained generalized logistic curves as well as the curve of the reported cumulative number of deaths (up to Day 300) are displayed in Fig. 1. The parameters of the derived curves are displayed in Table 1. In this Table, we also report: the values of the objective functions, the values of the root-mean-square errors (RMSE), and the CPU times.

We performed a Kolmogorov–Smirnov test for checking the hypothesis that the prediction errors are normally distributed. The results are displayed in Table 2. We observe that for both scenarios the null hypothesis (that is, the errors are normally distributed) cannot be rejected at the 5% significance level. Furthermore, we performed a hypothesis test for checking whether the mean error is zero. We found that, in both cases, it is rejected at the 5% significance level, but it cannot be rejected at the 1% significance level (see the the corresponding confidence intervals in Table 3).

To get a quantitative measure of the quality of the predictions, we computed for each prediction scenario (that is, $T = 120$ and 180, respectively), the relative deviations, which are defined as follows:

26 $$\begin{aligned} \text {Relative Deviation on Day } t = \frac{C_d(t)-\tilde{C}_d(t)}{\tilde{C}_d(t)}, \end{aligned}$$

for $t = T+1, \ldots , 215$, where 215 corresponds to the last day for which data is available (September 30, 2020). We found that the first scenario $(T = 120)$ exhibits a positive mean deviation of $7.22\%$, which indicates that the estimates are on the average slightly overestimated. However, the estimates are fairly robust since the maximum absolute deviation is $14.88\%$. On the other hand, the second scenario $(T = 180)$ exhibits a negative mean deviation of $-3.85\%$, and a remarkable maximum absolute deviation of $6.64\%$ only.

**Table 1:** Parameters values for the optimization approach.

${\mathrm {T}}$	$\mathrm {(K,Q,\lambda ,\nu )}$	$\mathrm {\sum \limits _{t}\epsilon _t^2}$	RMSE	CPU time (s)
$\mathrm {120}$	$K = 194,566$	920.7	9.93	182.349
	$$Q = 2000$$			
	$$\lambda = 0.0682$$			
	$$\nu = 1.2666$$			
$\mathrm {180}$	$K = 296,117$	1723.8	3.39	900.263
	$$Q = 131$$			
	$$\lambda = 0.0498$$			
	$$\nu = 0.7246$$

**Table 2:** KS test values for 95% confidence interval.

${\mathrm {T}}$	Hypothesis	P-value	KS statistic	CR value
$\mathrm {120}$	$H_0$	0.0752	0.1155	0.1225
$\mathrm {180}$	$H_0$	0.3447	0.0689	0.1003

**Table 3:** Confidence intervals (CI) characteristics.

${\mathrm {T}}$	Error STD	Error MEAN	95% CI	99% CI
$\mathrm {120}$	2.7186	0.5859	[0.094516, 1.0773]	$[-0.063572,1.2354]$
$\mathrm {180}$	3.0627	0.4982	[0.050747, 0.94561]	$[-0.089875,1.0862]$

Using (25), the first scenario $(T =120)$ yields an estimate value of the IFR that is equal to 0.11%. On the other hand, using much more available data, the second scenario $(T = 180)$ yields an estimate value that is equal to 0.068%. To the best of our knowledge, there is no published estimate of the IFR in Qatar, and therefore it is infeasible at this stage to get a rigorous assessment of the accuracy of our estimates. Nevertheless, it is possible to get some insights by comparing these values with those estimates that were recently published in the literature and that are based on data from antibody-prevalence studies. In particular, the authors in^60^ published the results of a nation-wide study in Spain that tested for antibodies in more than 61, 000 randomly selected individuals. This study provides estimates of the range of the age-specific IFR for severe acute respiratory syndrome coronavirus 2. The results of this study provide evidence that the IFR is nearly zero for anyone who is under forty and increases to 7.2% for people in their eighties (see Table 4).

We used Table 4 for deriving a “rough” estimate of overall IFR for Qatar. Toward this end, we considered the present distribution of the population of Qatar by age group^61^. This distribution is displayed in Table 5. Combining the data in Tables 4 and 5, we derived an overall IFR for Qatar that is equal to 0.086%, which is pretty close to the estimate value that was obtained for $T = 180$. At this stage, it is worth emphasizing that it is reasonable to expect that differences between countries in the IFR estimates may exist because the fatality risk is related, among other factors, to the underlying public health system.

**Table 4:** IFR by age^62^.

Age group	IFR by age (%)
0–9	0
10–19	0
20–29	0.01
30–39	0.03
40–49	0.07
50–59	0.3
60–69	1
70–79	3.4
≥ 80	7.2

**Table 5:** Distribution of Qatar population^61^.

Age group	Population in Qatar (in 1000 s)
0–9	287.51
10–19	197.14
20–29	695.37
30–39	911.83
40–49	458.65
50–59	179.55
60–69	52.87
70–79	12.79
≥ 80	3.47
Total	2799.18

**Figure 2:**
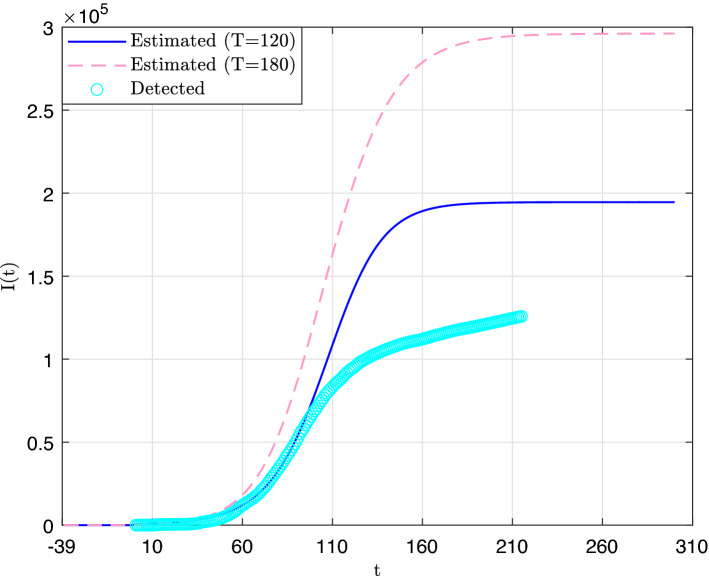
Estimate total number of infected cases curves.

**Figure 3:**
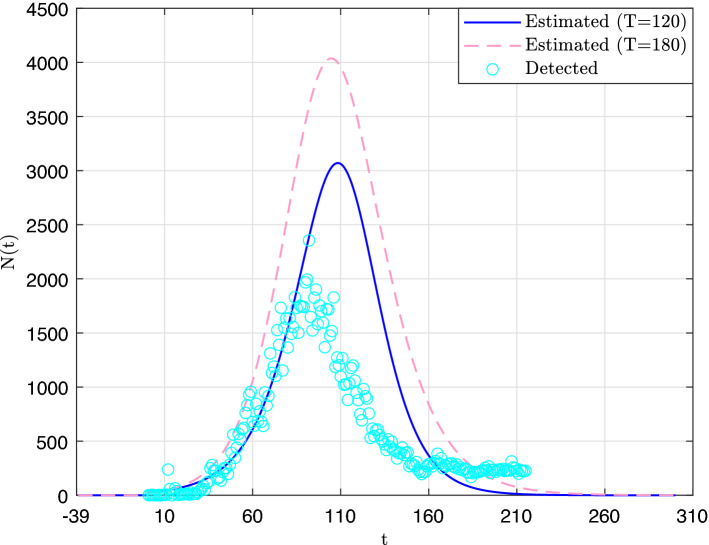
Estimate daily number of infected cases curves.

In Figs. 2 and 3 are displayed the curves of the total number of infected cases and the daily number of infected cases, respectively, along with the reported detected cases. Both figures suggest that the number of detected cases (through RT-PCR tests) largely underestimates the actual spread of the outbreak. Also, we observe that in both cases, the estimate values have generally increased between the first prediction (Day 120) and the second one (Day 180). In particular, the values of parameter *K*, which corresponds to the asymptotic limit of *I*(*t*) was first estimated to 195,000 and then to 296,000. This latter estimate suggests that by the end of the outbreak about 10.5% of the population of Qatar would have been infected by the SARS-Cov-2. At this point it is worth mentioning that sampling-based methods for estimating seroprevalence of anti-SARS-CoV-2 antibodies in a population have been used elsewhere by many authors. In particular, a study that was carried out on a large sample of randomly selected adult patients receiving dialysis in the US found evidence that 9.3% of the US adult population has been infected so far^3^. On the other hand, a second study^63^ that was carried out in the region of Geneva, Switzerland (which was heavily affected by the first wave of the outbreak), found a seroprevalence ranging on different weeks between 4.8 and 10.9%.

**Figure 4:**
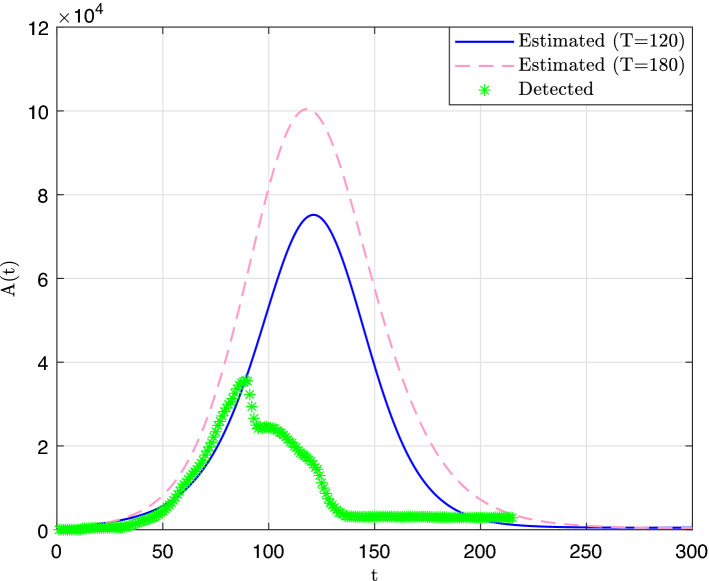
Estimate daily number of active cases curves.

Using (10) and (13), we derived estimates of the number of active cases. The obtained curves are displayed in Fig. 4. Here again, we observe that the number of detected active cases are much smaller than the predicted values, and that the estimate values of Day 180 are significantly larger than were obtained on Day 120.

## Conclusion

Keeping in mind that the proposed approach is intended to be used primarily for forecasting of the number of deaths and estimation of the death rate from infection, we believe that it offers remarkable advantages over similar approaches, but also has some limitations. It is a glaring fact that the vast majority of the modeling approaches that have been proposed so far for modeling and forecasting the spread of COVID-19 rely heavily on data and parameters that are difficult to know precisely. Indeed, these models require information on many parameters that pertain to mobility and behavior of the population, the reproduction number $R_0$, the current number of infections, the infection fatality ratio, as well as the fraction of asymptomatic (contagious) individuals, to quote just a few. Often, a small variation in any of these input data and the model can provide forecasts that are off by orders of magnitude. In contrast, the proposed approach is based solely on the number of deaths, which is both much more precise and easier to obtain. At this point, it should be noted that the number of observed deaths is rarely used in the literature to predict the number of deaths. However, whenever this number is used, other parameters are also used. For example, the authors of^64^ have developed an interesting SIR model to estimate the number of new COVID-19 infections, as well as the number of people likely to die. However, unlike our approach, they also rely on the infection fatality rate (IFR) for which accurate estimate is still unknown. Here we emphasize that, in our approach, the IFR is an output of the model calculations and not an input. On the other hand, an obvious limitation of our approach is that it assumes that the spread of the epidemic is a stationary process. That is, we assume that no new viral variants have appeared, nor that new epidemic control measures or vaccination campaigns have been implemented. Furthermore, a second limitation is that it cannot be useful for performing a what-if-analysis. Which means that it cannot help answer questions such as “what will be the impact of social distancing?” or “what will be the impact of the school closures?”

We anticipate that if the proposed approach is run on a periodic basis (as new data accumulates), then it will prove useful for public health authorities for making informed decisions and thus mitigating the impact of the outbreak.

Finally, we used in this study (for the sake of convenience) the PSO code that is embedded within MATLAB. However, it is likely that some recent and much sophisticated PSO variants (including those presented in^51–53^) might prove much effective and thus would probably yield better estimate values. This is a first future research direction that we recommend investigating. Furthermore, we recommend as a future research direction to use the proposed approach for deriving estimates of the IFR for different countries and to develop a regression analysis for investigating the influence of socioeconomic, demographic, climatological and environmental factors on the spread on COVID-19.

## Data Availability

The data used in the current study are publicly available in : Coronavirus disease 2019 (COVID-19) statistics. https://www.data.gov.qa/explore/dataset/covid-19-cases-in-qatar/table/?sort=date. Worldometer coronavirus. https://www.worldometers.info/coronavirus/country/qatar/. Population of Qatar in 2019, by age group (in 1,000s). https://www.statista.com/statistics/724145/qatar-population-age-group/.

## References

[1] Rathore V, Galhotra A, Pal R, Sahu KK (2020). COVID-19 pandemic and children: A review. J. Pediatr. Pharmacol. Ther..

[2] Sahu KK, Kumar R (2020). Current perspective on pandemic of COVID-19 in the United States. J. Fam. Med. Primary Care.

[3] Anand S (2020). Prevalence of SARS-CoV-2 antibodies in a large nationwide sample of patients on dialysis in the USA: A cross-sectional study. Lancet J..

[4] Valvo PS (2020). A bimodal lognormal distribution model for the prediction of COVID-19 deaths. Appl. Sci..

[5] Abdy M, Side S, Annas S, Nur W, Sanusi W (2021). An SIR epidemic model for COVID-19 spread with fuzzy parameter: The case of Indonesia. Adv. Diff. Equ..

[6] Buhat CAH (2021). A mathematical model of COVID-19 transmission between frontliners and the general public. Netw. Model. Anal. Health Inf. Bioinform..

[7] Mohammad M, Trounev A, Cattani C (2021). The dynamics of COVID-19 in the UAE based on fractional derivative modeling using Riesz wavelets simulation. Adv. Diff. Equ..

[8] Alvarez MM, González-González E, de Santiago GT (2021). Modeling COVID-19 epidemics in an Excel spreadsheet to enable first-hand accurate predictions of the pandemic evolution in urban areas. Sci. Rep..

[9] Wijaya KP (2021). An epidemic model integrating direct and fomite transmission as well as household structure applied to COVID-19. J. Math. Ind..

[10] Oud MAA (2021). A fractional order mathematical model for COVID-19 dynamics with quarantine, isolation, and environmental viral load. Adv. Diff. Equ..

[11] Jarndal, A., Husain, S., Zaatar, O., Gumaei, T. A. & Hamadeh, A. GPR and ANN based prediction models for COVID-19 death cases. In *2020 International Conference on Communications, Computing, Cybersecurity, and Informatics (CCCI)*, 1–5, 10.1109/CCCI49893.2020.9256564 (2020).

[12] Bohk-Ewald C, Dudel C, Myrskylä M (2020). A demographic scaling model for estimating the total number of COVID-19 infections. Int. J. Epidemiol..

[13] Coccia M (2021). Effects of the spread of COVID-19 on public health of polluted cities: Results of the first wave for explaining the dejà vu in the second wave of COVID-19 pandemic and epidemics of future vital agents. Environ. Sci. Pollut. Res..

[14] Coccia M (2020). An index to quantify environmental risk of exposure to future epidemics of the COVID-19 and similar viral agents: Theory and practice. Environ. Res..

[15] Diao Y (2021). Influence of population density, temperature, and absolute humidity on spread and decay durations of COVID-19: A comparative study of scenarios in China, England, Germany, and Japan. One Health.

[16] Coccia M (2021). The relation between length of lockdown, numbers of infected people and deaths of Covid-19, and economic growth of countries: Lessons learned to cope with future pandemics similar to Covid-19 and to constrain the deterioration of economic system. Sci. Total Environ..

[17] Coccia M (2021). The impact of first and second wave of the COVID-19 pandemic in society: Comparative analysis to support control measures to cope with negative effects of future infectious diseases. Environ. Res..

[18] Coccia M (2021). High health expenditures and low exposure of population to air pollution as critical factors that can reduce fatality rate in COVID-19 pandemic crisis: A global analysis. Environ. Res..

[19] Zhao S (2021). Inferencing superspreading potential using zero-truncated negative binomial model: Exemplification with COVID-19. BMC Med. Res. Methodol..

[20] Weinberger DM (2020). Estimation of excess deaths associated with the COVID-19 pandemic in the United States, March to May 2020. JAMA Intern. Med..

[21] Aburto JM (2021). Estimating the burden of the COVID-19 pandemic on mortality, life expectancy and lifespan inequality in England and Wales: A population-level analysis. J. Epidemiol. Commun. Health.

[22] Choi BCK, Pak AWP (2003). A simple approximate mathematical model to predict the number of severe acute respiratory syndrome cases and deaths. J. Epidemiol. Commun. Health.

[23] Naheed A, Singh M, Lucy D (2014). Numerical study of SARS epidemic model with the inclusion of diffusion in the system. Appl. Math. Comput..

[24] Liang K (2020). Mathematical model of infection kinetics and its analysis for COVID-19, SARS and MERS. Infect. Genet. Evol..

[25] Xia Z-Q (2015). Modeling the transmission dynamics of Ebola virus disease in Liberia. Sci. Rep..

[26] Al-Asuoad N, Alaswad S, Rong L, Shillor M (2016). Mathematical model and simulations of MERS outbreak: Predictions and implications for control measures. Biomath.

[27] Huang H, Chen Y, Ma Y (2021). Modeling the competitive diffusions of rumor and knowledge and the impacts on epidemic spreading. Appl. Math. Comput..

[28] Wang L, Liu Z, Guo C, Li Y, Zhang X (2021). New global dynamical results and application of several SVEIS epidemic models with temporary immunity. Appl. Math. Comput..

[29] Singh S (2020). Study of ARIMA and least square support vector machine (LS-SVM) models for the prediction of SARS-CoV-2 confirmed cases in the most affected countries. Chaos Solitons Fractals.

[30] Ciufolini I, Paolozzi A (2020). Mathematical prediction of the time evolution of the COVID-19 pandemic in Italy by a Gauss error function and Monte Carlo simulations. Eur. Phys. J. Plus.

[31] Salje H (2020). Estimating the burden of SARS-CoV-2 in France. Science.

[32] Salgotra R, Gandomi M, Gandomi AH (2020). Time series analysis and forecast of the COVID-19 pandemic in India using genetic programming. Chaos Solitons Fractals.

[33] Postnikov EB (2020). Estimation of COVID-19 dynamics on a back-of-envelope: Does the simplest SIR model provide quantitative parameters and predictions?. Chaos Solitons Fractals.

[34] Verity R (2020). Estimates of the severity of coronavirus disease 2019: A model-based analysis. Lancet Infect. Dis..

[35] Calafiore, G. C., Novara, C. & Possieri, C. A modified SIR model for the COVID-19 contagion in Italy. 1–6 (2020). https://arxiv.org/abs/2003.14391. 10.1016/j.arcontrol.2020.10.005 PMC7587010 33132739

[36] Sanchez-Caballero S, Selles MA, Peydro MA, Perez-Bernabeu E (2020). An efficient COVID-19 prediction model validated with the cases of China, Italy and Spain: Total or partial lockdowns?. J. Clin. Med..

[37] Peng, L., Yang, W., Zhang, D., Zhuge, C. & Hong, L. Epidemic analysis of COVID-19 in China by dynamical modeling. 1–11 (2020). https://arxiv.org/abs/2002.06563.

[38] Zhu H (2020). Transmission dynamics and control methodology of COVID-19: A modeling study. Appl. Math. Model..

[39] Liu J, Wang L, Zhang Q, Yau S-T (2020). The dynamical model for COVID-19 with asymptotic analysis and numerical implementations. Appl. Math. Model..

[40] Rafiq M, Macías-Díaz RA, Ahmed N (2021). Design of a nonlinear model for the propagation of COVID-19 and its efficient nonstandard computational implementation. Appl. Math. Model..

[41] Kudryashov NA, Chmykhov M, Vigdorowitsch M (2021). Analytical features of the SIR model and their applications to COVID-19. Appl. Math. Model..

[42] Hsieh Y-H, Lee J-Y, Chang H-L (2004). SARS epidemiology modeling. Emerg. Infect. Dis..

[43] Hsieh, Y.-H. Richards model: A simple procedure for real-time prediction of outbreak severity. In *Modeling and Dynamics of Infectious Diseases*, 216–236 (World Scientific, 2009) 10.1142/9789814261265_0009.

[44] Hsieh Y-H, Ma S (2009). Intervention measures, turning point, and reproduction number for dengue. Am. J. Trop. Med. Hygiene.

[45] Hsieh Y-H, Chen CWS (2009). Turning points, reproduction number, and impact of climatological events for multi-wave dengue outbreaks. Trop. Med. Int. Health.

[46] Pelinovsky E, Kurkin A, Kurkina O, Kokoulina M, Epifanova A (2020). Logistic equation and COVID-19. Chaos Solitons Fractals.

[47] Lee SY, Lei B, Mallick B (2020). Estimation of COVID-19 spread curves integrating global data and borrowing information. PLOS ONE.

[48] Wu K, Darcet D, Wang Q, Sornette D (2020). Generalized logistic growth modeling of the COVID-19 outbreak: comparing the dynamics in the 29 provinces in China and in the rest of the world. Nonlinear Dyn..

[49] Jukić D, Scitovski R (1996). The existence of optimal parameters of the generalized logistic function. Appl. Math. Comput..

[50] Kennedy, J. & Eberhart, R. Particle Swarm Optimization. In *Proceedings of ICNN’95 - International Conference on Neural Networks*, vol. 4, 1942–1948, 10.1109/ICNN.1995.488968 (1995).

[51] Tsoulos IG, Tzallas A, Karvounis E (2020). Improving the PSO method for global optimization problems. Evol. Syst..

[52] Wang C-F, Liu KA (2016). Algorithm, novel particle swarm optimization & for global optimization. Comput. Intell. Neurosci..

[53] Koyuncu H, Ceylan R (2019). A PSO based approach: Scout particle swarm algorithm for continuous global optimization problems. J. Comput. Des. Eng..

[54] Abdallah, M. A. & Nafea, M. PSO-Based SEIQRD Modeling and Forecasting of COVID-19 Spread in Italy. In *2021 IEEE 11th IEEE Symposium on Computer Applications Industrial Electronics (ISCAIE)*, 71–76, 10.1109/ISCAIE51753.2021.9431836 (2021).

[55] Zhan, C., Wu, Z., Wen, Q., Gao, Y. & Zhang, H. Optimizing Broad Learning System Hyper-parameters through Particle Swarm Optimization for Predicting COVID-19 in 184 Countries. In *2020 IEEE International Conference on E-health Networking, Application & Services (HEALTHCOM)*, 1–6, 10.1109/HEALTHCOM49281.2021.9399020 (2021).

[56] Zreiq R (2020). Generalized Richards model for predicting COVID-19 dynamics in Saudi Arabia based on particle swarm optimization Algorithm. AIMS Public Health.

[57] Lauer SA (2019). (COVID-19) from publicly reported confirmed cases: Estimation and application. Ann. Intern. Med..

[58] Coronavirus disease 2019 (COVID-19) statistics. https://www.data.gov.qa/explore/dataset/covid-19-cases-in-qatar/table/?sort=date.

[59] Worldometer coronavirus. https://www.worldometers.info/coronavirus/country/qatar/.

[60] Pastor-Barriuso R (2020). Infection fatality risk for SARS-CoV-2: A nationwide seroepidemiological study in the non-institutionalized population of Spain. MedRxiv.

[61] Population of Qatar in 2019, by age group (in 1,000s). https://www.statista.com/statistics/724145/qatar-population-age-group/ (2020).

[62] Mallapaty S (2020). The coronavirus is most deadly if you are older and male. Nature.

[63] Stringhini S (2020). Seroprevalence of anti-SARS-CoV-2 IgG antibodies in Geneva, Switzerland (SEROCoV-POP): A population-based study. Lancet J..

[64] Johndrow, J., Lum, K. & Ball, P. Estimating SARS-CoV-2-positive Americans using deaths-only data. 1–15 (2020). https://arxiv.org/abs/2004.02605v1.

